# Synthesis, biological evaluation and molecular docking studies of 6-(4-nitrophenoxy)-1*H*-imidazo[4,5-b]pyridine derivatives as novel antitubercular agents: future DprE1 inhibitors

**DOI:** 10.1186/s13065-018-0515-1

**Published:** 2018-12-19

**Authors:** Jineetkumar Gawad, Chandrakant Bonde

**Affiliations:** Department of Pharmaceutical Chemistry, School of Pharmacy & Technology Management, SVKM’s NMIMS, Shirpur Campus, Dhule, 425 405 India

**Keywords:** Tuberculosis, Imidazopyridine derivatives, DprE1 inhibitors, Antitubercular activity

## Abstract

Tuberculosis is an air-borne disease, mostly affecting young adults in their productive years. Here, Ligand-based drug design approach yielded a series of 23 novel 6-(4-nitrophenoxy)-1*H*-imidazo[4,5-b]pyridine derivatives. The required building block of imidazopyridine was synthesized from commercially available 5,5-diaminopyridine-3-ol followed by four step sequence. Derivatives were prepared using various substituted aromatic aldehydes. All the synthesized analogues were characterized using NMR, Mass analysis and also screened for in vitro antitubercular activity against *Mycobacterium tuberculosis* (H_37_Rv). Four compounds, **5c** (MIC-0.6 μmol/L); **5g** (MIC-0.5 μmol/L); **5i** (MIC-0.8 μmol/L); and **5u** (MIC-0.7 μmol/L) were identified as potent analogues. Drug receptor interactions were studied with the help of ligand docking using maestro molecular modeling interphase, Schrodinger. Here, computational studies showed promising interaction with other residues with good score, which is novel finding than previously reported. So, these compounds may exhibit in vivo DprE1 inhibitory activity.

**Electronic supplementary material:**

The online version of this article (10.1186/s13065-018-0515-1) contains supplementary material, which is available to authorized users.

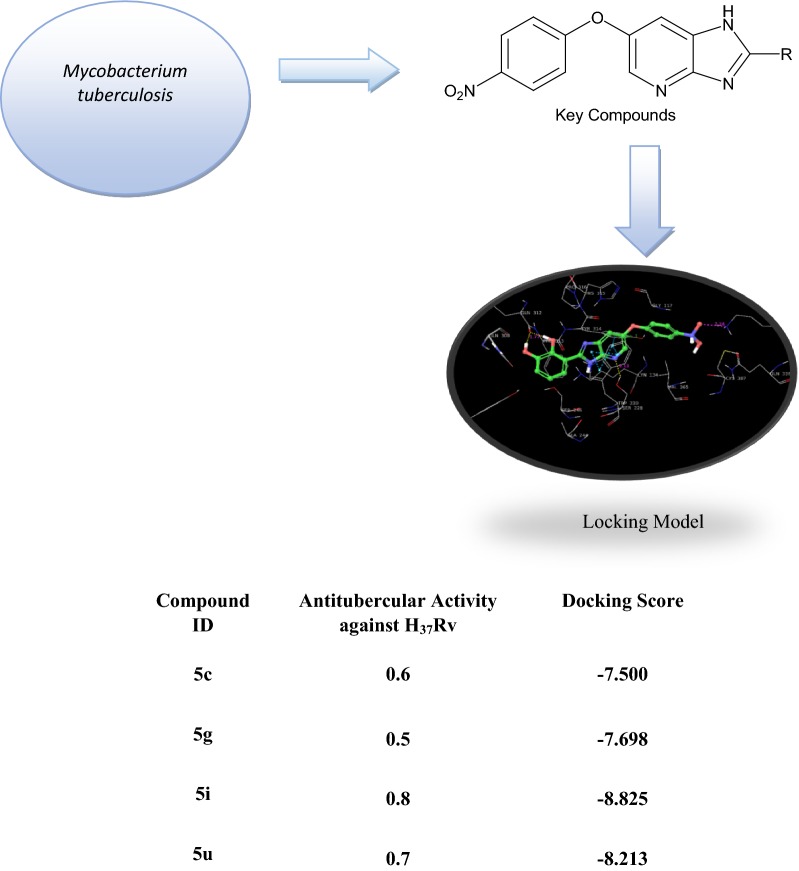

## Introduction

Tuberculosis is major threat for mankind from past several decades. Tuberculosis is the leading cause of death from infectious diseases [1]. Although the number of tuberculosis cases decreased during the twentieth century, the emergence of HIV and the incidence of multiple-drug resistance (MDR) have increased the difficulty of treating many new cases. Despite of the efforts taken to improve the outcome of tuberculosis care, the discovery of new antibiotics against the causative agent is not in a race of expected progress [2, 3]. With this, new and more effective molecules with novel mechanism of action are required to discover which may shorten the treatment, improve patient adherence, and reduce the appearance of resistance [4].

Furthermore, *Mycobacterium tuberculosis* (*M. tuberculosis*) has also proven one of the world’s most dreadful human pathogen because of its ability to persist inside humans for longer time period in a clinically inactive state. Roughly 95% of the general population who infected (33% of the worldwide population) built up an inert infection [5, 6]. The current available vaccine, *Mycobacterium bovis* Bacillus Calmette–Guerin (BCG). *M. tuberculosis* stimulates a solid response, however it has ability to oppose the body’s activities to kill it and regardless of the possibility of underlying disease is effectively controlled. The discovery of drugs with novel mechanism of action is required because of the expanding number of MDR, which are strains of *M. tuberculosis* that are resistant to both isoniazid and rifampicin (first line therapy), with or without protection from different medications, broadly extensively drug resistance (XDR) and MDR strains additionally resistant to any fluoroquinolone and any of the second-line against TB injectable medications (amikacin, kanamycin, or capreomycin). Imidazopyridine derivatives are very important, versatile motifs with significant applications in medicinal chemistry [7–9].

The imidazopyridine scaffold was found in a number of marketed drug formulations and drug candidates such as antiulcer-zolimidine [10] and tenatoprazole [11–13], sedative-zolpidem [14], anxiolytic-saripidem [15] and necopidem [16, 17], analgesic and antipyretic-microprofen [18], cardiotonic-olprinone [19, 20], anti-tumour-3-deazaneplanocin A [21, 22]. Fortunately, 3-deazaneplanocin A was also found to be effective for the treatment against Ebola virus disease [23–26]. In addition, compounds containing the moiety imidazopyridine have significant biological applications such as antimycobacterial, anticoccidial, antimicrobial [27–34].

In other words, the therapeutic application of imidazopyridine is not restricted, and need to explore to the fullest for the betterment of mankind. Here, we are looking forward to uncover the potential of 1*H*-imidazo[4,5-b]pyridine nucleus as a biological agent, hence, we thought to synthesize 6-(4-nitrophenoxy)-2-substituted-1*H*-imidazo[4,5-b]pyridine derivatives. Purposely 4-nitrophenoxy substitution was chosen on 6th position of 1*H*-imidazo[4,5-b]pyridine ring because it was proved that the nitro containing compounds shown binding with cys387 residue of DprE1 enzyme protein.

Reports of World Health Organisation (WHO) in past couple of years pointed out that, the global burden of tuberculosis is increasing drastically across the globe. With this threatening scenario of tuberculosis infection, it’s a strict need to search promising drugs which will effectively kill the *mycobacterium* within short duration of time. Here, we have made an attempt to synthesized novel compounds of imidazopyridine series for antitubercular activity, which may target particularly decaprenyl-phosphoryl-ribose 2′-epimerase (DprE1) enzyme (DprE1 is a novel target for which no drug is available in market till date) in search of novel lead for antitubercular drug discovery to serve the society.

## Experimental

### Chemistry

All the chemicals were obtained from Sigma Aldrich, Germany, Merk India, Rankem India, Loba Chemi, India, Signichem laboratories, India. Melting points (m.p.) were detected with open capillaries using Veego Melting point apparatus, Mumbai India and are uncorrected. IR spectra were recorded on IR Affinity-1S (FTIR, Schimadzu, Japan) spectrophotometer. ^1^H and ^13^C NMR was obtained using a JEOL, JAPAN ECZR Series 600 MHz NMR Spectrometer using tetramethylsilane (TMS) as internal standard. All chemical shift values were recorded as *δ* (ppm), coupling constant value *J* was measured in hertz, the peaks are presented as s (singlet), d (doublet), t (triplet), dd (double doublet), m (multiplet). The purity of compounds was controlled by thin layer chromatography (Qualigens Fine Chemicals Mumbai, silica gel, GF-254).

### General procedure for synthesis

**Scheme 1:**
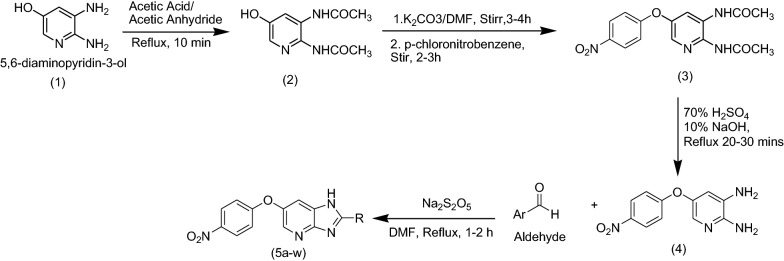
Synthesis of 6-(4-nitrophenoxy)-1*H*-imidazo[4,5-b]pyridine derivatives

5,6-Diaminopyridine-3-ol and different substituted aromatic aldehydes were commercially available. The process of four step reaction sequence was initiated with acetylation of 5,6-diaminopyridine-3-ol **1** which on reaction with acetic anhydride forms compound **2** by nucleophilic substitution reaction [35]. To increase the reactivity of –OH, the hydroxyl group, it is converted to its potassium salt by stirring compound **2** [36] with K_2_CO_3_ in dimethylformamide (DMF) for 3–4 h and then, *p*-chloronitrobenzene diluted in DMF (1:1) was added drop-wise for 1 h [37]. Again reaction mixture was stirred for 2–3 h to obtained compound **3**. Further, the reactions mixture was poured in cold 10% sodium hydroxide [38, 39]. The compound **4** was precipitated out which further recrystallized by ethanol [40, 41]. Compound **4** on reaction with different substituted aromatic aldehydes (Table 1) in presence of Na_2_S_2_O_5_ yielded compound **5** derivatives (Scheme 1).

**Table 1:**
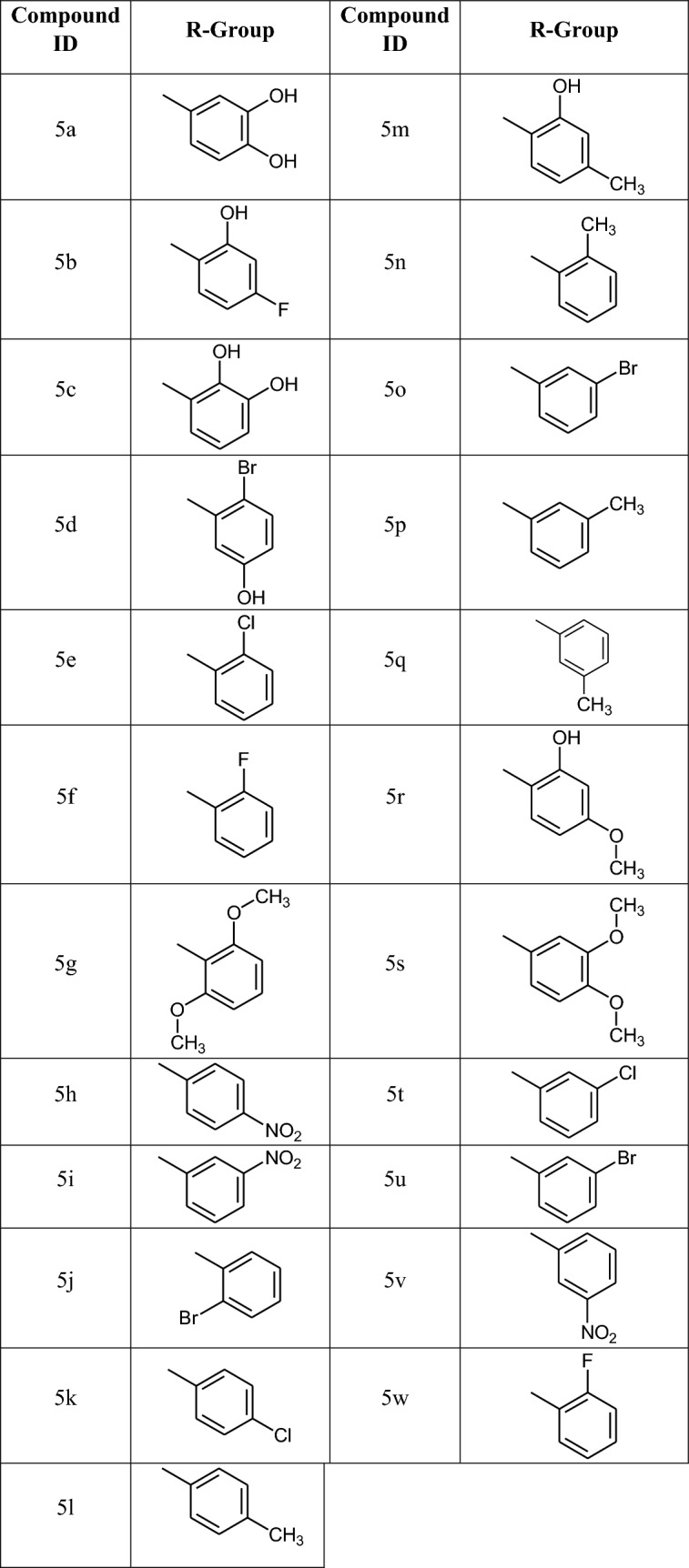
Synthesis of compounds from **5a–w**

**1**: 5,6-diaminopyridine-3-ol. IR v = 1390 cm^−1^ (C–N str), 1780 cm^−1^ (aromatic ring), 3320 cm^−1^ (O–H str), ^1^H NMR: (600 MHz, DMSO) δ 6.4 (1H, d, J = 2.7 Hz), 7.7 (1H, d, J = 2.7 Hz).^13^C NMR (100 MHz, DMSO) δ (ppm) 100.9, 135.2, 140.4, 153.2. MS m/z: calcd for C_5_H_7_N_3_O found 125.13 (M–H)^−^: 124.61.

**2**: *N*-(3-acetamido-5-hydroxypyridin-2-yl)acetamide. IR v = 1670 cm^−1^ (C–O str), 1670 cm^−1^ (aromatic ring), 3420 cm^−1^ (O–H str), ^1^H NMR: (600 MHz, DMSO) δ 2.7–2.9 (6H, m), 7.2 (1H, d, J = 2.3 Hz), 7.8 (1H, d, J = 2.3 Hz).^13^C NMR (100 MHz, DMSO) δ (ppm) 23.9, 100.9, 121.7, 135.2, 142.5, 153.2, 168.7. MS m/z: calcd for C_9_H_11_N_3_O_3_ found 209.20 (M–H)^−^: 208.65.

**4**: 5-(4-nitrophenoxy)pyridine-2,3-diamine. IR v = 1540 cm^−1^ (N–O str), 1680 cm^−1^ (C–O ether), 1530, 1620 cm^−1^ (aromatic ring), 1440 cm^−1^ (C–N str), 1H NMR: (600 MHz, DMSO) δ 6.8 (1H, d, J = 2.8 Hz), 7.2–7.3 (4H, m), 7.8 (1H, d, J = 2.8 Hz).^13^C NMR (100 MHz, DMSO) δ (ppm) 100.9, 116.9, 124.5, 135.2, 140.4, 143.2, 151.5, 163.8. MS m/z: calcd for C_11_H_12_N_4_O_3_ found 248.23 (M–H)^−^: 247.63.

**5a**: 4-[6-(4-nitrophenoxy)-1*H*-imidazo[4,5-b]pyridin-2-yl]benzene-1,2-diol. Yield: 32%. M.P. 140 °C–142 °C. IR v = 1540 cm^−1^ (N–O str), 1150 cm^−1^ (C–O ether), 1480, 1550, 1690, 1740 cm^−1^ (aromatic ring), 3470 cm^−1^ (O–H str), ^1^H NMR: (600 MHz, DMSO) δ 4.0 (2H, s), 6.9 (1H, dd, J = 8.9, 0.4 Hz), 7.2 (2H, dd, J = 8.4, 1.5 Hz), 7.3 (1H, d, J = 1.8 Hz), 7.4 (1H, dd, J = 8.9, 1.8 Hz), 7.9 (1H, d, J = 1.6 Hz), 8.0 (2H, dd, J = 8.4, 1.9 Hz), 8.6 (1H, d, J = 1.6 Hz).^13^C NMR (100 MHz, DMSO) δ (ppm) 40.4, 115.3, 119.7, 123.5, 126.8, 130.1, 137.8, 145.3, 146.6, 151.2. MS m/z: calcd for C_18_H_12_N_4_O_5_ found 364.08 (M–H)^−^: 363.53.

**5b**: 5-fluoro-2-[6-(4-nitrophenoxy)-1*H*-imidazo[4,5-b]pyridin-2-yl]phenol. Yield: 45%. M.P. 157 °C–159 °C. IR v = 1420 cm^−1^ (N–O str), 1190 cm^−1^ (C–O ether), 1430, 1540, 1890 cm^−1^ (aromatic ring), 3220 cm^−1^ (O–H str), ^1^H NMR: (600 MHz, DMSO) δ 4.0 (2H, s), 6.3 (1H, d, J = 1.6 Hz), 6.4 (1H, dd, J = 8.5, 1.6 Hz), 7.2 (2H, dd, J = 8.5, 1.5 Hz), 7.6 (1H, d, J = 8.5 Hz), 7.9 (1H, d, J = 1.6 Hz), 8.0 (2H, dd, J = 8.5, 1.9 Hz), 8.7 (1H, d, J = 1.6 Hz).^13^C NMR (100 MHz, DMSO) δ (ppm) 40.4, 100.5, 113.4, 117.2, 127.8, 140.4, 148.9, 158.7, 162.2. MS m/z: calcd for C_18_H_11_FN_4_O_4_ found 366.07 (M–H)^−^: 365.37.

**5c**: 3-[6-(4-nitrophenoxy)-1*H*-imidazo[4,5-b]pyridin-2-yl]benzene-1,2-diol. Yield: 30%. M.P. 148 °C–150 °C. IR v = 1380 cm^−1^ (N–O str), 1120 cm^−1^ (C–O ether), 1490, 1630, 1770 cm^−1^ (aromatic ring), 3360 cm^−1^ (O–H str), ^1^H NMR: (600 MHz, DMSO) δ 4.0 (2H, s), 6.9 (1H, dd, J = 8.0, 1.3 Hz), 7.1–7.3 (3H, m), 7.3 (1H, dd, J = 7.8, 1.3 Hz), 7.9 (1H, d, J = 1.6 Hz), 8.0 (2H, dd, J = 8.4, 1.9 Hz), 8.6 (1H, d, J = 1.6 Hz). ^13^C NMR (100 MHz, DMSO) δ (ppm) 40.4, 115.6, 126.3, 137.8, 145.2, 146.5, 152.3. MS m/z: calcd for C_18_H_12_N_4_O_5_ found 364.09 (M–H)^−^: 363.49.

**5d**: 4-bromo-3-[6-(4-nitrophenoxy)-1*H*-imidazo[4,5-b]pyridin-2-yl]phenol. Yield: 49%. M.P. 135 °C–137 °C. IR v = 1470 cm^−1^ (N–O str), 1140 cm^−1^ (C–O ether), 1580, 1630, 1850 cm^−1^ (aromatic ring), 3320 cm^−1^ (O–H str), ^1^H NMR: (600 MHz, DMSO) δ 4.0 (2H, s), 6.9 (1H, d, J = 8.2 Hz), 7.0 (1H, dd, J = 8.2, 2.7 Hz), 7.2 (2H, dd, J = 8.4, 1.5 Hz), 7.3 (1H, d, J = 2.7 Hz), 7.9 (1H, d, J = 1.6 Hz), 8.0 (2H, dd, J = 8.4, 1.9 Hz), 8.6 (1H, d, J = 1.6 Hz). ^13^C NMR (100 MHz, DMSO) δ (ppm) 40.4, 115.6, 129.9, 138.6, 148.9, 158.7. MS m/z: calcd for C_18_H_11_BrN_4_O_4_ found 427.21 (M–H)^−^: 426.65.

**5e**: 2-(2-chlorophenyl)-6-(4-nitrophenoxy)-1*H*-imidazo[4,5-b]pyridine. Yield: 52%. M.P. 152 °C–154 °C. IR v = 1420 cm^−1^ (N–O str), 1160 cm^−1^ (C–O ether), 1620, 1740, 1730 cm^−1^ (aromatic ring), 3420 cm^−1^ (O–H str) ^1^H NMR: (600 MHz, DMSO) δ 4.1 (2H, s), 7.1 (1H, d, J = 8.1 Hz), 7.2-7.4 (3H, m), 7.9 (1H, dd, J = 7.6, 1.7 Hz), 8.0–8.7 (3H, m), 8.7 (1H, d, J = 1.6 Hz). ^13^C NMR (75 MHz, DMSO) δ (ppm) 40.4, 113.4, 126.8, 140.4, 158.7. MS m/z: calcd for C_18_H_11_ClN_4_O_3_ found 366.76 (M–H)^−^: 365.57.

**5f**: 2-(2-fluorophenyl)-6-(4-nitrophenoxy)-1*H*-imidazo[4,5-b]pyridine. Yield: 43%. M.P. 137 °C–139 °C. IR v = 1370 cm^−1^ (N–O str), 1190 cm^−1^ (C–O ether), 1710, 1770, 1780 cm^−1^ (aromatic ring), 3450 cm^−1^ (O–H str) ^1^H NMR: (600 MHz, DMSO) δ 7.2–7.5 (3H, m), 7.3–7.5 (2H, m), 7.6 (1H, d, J = 1.7 Hz), 7.9 (1H, dd, J = 7.6, 1.7 Hz), 8.1 (2H, dd, J = 8.3, 2.1 Hz), 8.4 (1H, d, J = 1.7 Hz). ^13^C NMR (100 MHz, DMSO) δ (ppm) 100.9, 114.2, 127.5, 140.4, 152.3, 156.0, 160.4. MS m/z: calcd for C_18_H_11_FN_4_O_3_ found 350.09 (M–H)^−^: 349.57.

**5g**: 2-(2,6-dimethoxyphenyl)-6-(4-nitrophenoxy)-1*H*-imidazo[4,5-b]pyridine. Yield: 46%. M.P. 128 °C–130 °C. IR v = 1510 cm^−1^ (N–O str), 1120 cm^−1^ (C–O ether), 1650, 1760, 1660 cm^−1^ (aromatic ring), 3520 cm^−1^ (O–H str) ^1^H NMR: (600 MHz, DMSO) δ 3.8 (6H, s), 6.9 (2H, dd, J = 8.1, 1.2 Hz), 7.3 (2H, dd, J = 8.4, 1.3 Hz), 7.4–7.5 (2H, m), 8.1 (2H, dd, J = 8.3, 2.1 Hz), 8.2 (1H, d, J = 1.7 Hz). ^13^C NMR (100 MHz, DMSO) δ (ppm) 55.8, 100.9, 117.2, 130.6, 140.4, 151.2, 156.0. MS m/z: calcd for C_20_H_16_N_4_O_5_ found 392.12 (M–H)^−^: 391.56.

**5h**: 6-(4-nitrophenoxy)-2-(4-nitrophenyl)-1*H*-imidazo[4,5-b]pyridine. Yield: 26%. M.P. 164 °C–166 °C. IR v = 1360 cm^−1^ (N–O str), 1175 cm^−1^ (C–O ether), 1750, 1770, 1790 cm^−1^ (aromatic ring), 3130 cm^−1^ (O–H str) ^1^H NMR: (600 MHz, DMSO) δ 7.3 (2H, dd, J = 8.4, 1.4 Hz), 7.8 (1H, d, J = 1.6 Hz), 7.9 (2H, dd, J = 8.8, 1.6 Hz), 8.1–8.2 (4H, m), 8.7 (1H, d, J = 1.6 Hz). ^13^C NMR (100 MHz, DMSO) δ (ppm) 100.9, 115.0, 126.1, 135.2, 145.4, 156.0. MS m/z: calcd for C_18_H_11_N_5_O_5_ found 377.09 (M–H)^−^: 376.47.

**5i**: 6-(4-nitrophenoxy)-2-(3-nitrophenyl)-1*H*-imidazo[4,5-b]pyridine Yield: 29%. M.P. 149 °C–151 °C. IR v = 1380 cm^−1^ (N–O str), 1160 cm^−1^ (C–O ether), 1610, 1720, 1770 cm^−1^ (aromatic ring), 3490 cm^−1^ (O–H str) ^1^H NMR: (600 MHz, DMSO) δ 7.3 (2H, dd, J = 8.4, 1.4 Hz), 7.6 (1H, dd, J = 8.7, 7.6 Hz), 7.8 (1H, d, J = 1.6 Hz), 8.0 (1H, dd, J = 7.9, 1.6 Hz), 8.1–8.2 (4H, m) 8.7 (1H, d, J = 1.6 Hz). ^13^C NMR (100 MHz, DMSO) δ (ppm) 100.9, 117.2, 126.9, 140.4, 156.0. MS m/z: calcd for C_18_H_11_N_5_O_5_ found 377.20 (M–H)^−^: 376.59.

**5j**: 2-(2-bromophenyl)-6-(4-nitrophenoxy)-1*H*-imidazo[4,5-b]pyridine. Yield: 33%. M.P. 170 °C–172 °C. IR v = 1490 cm^−1^ (N–O str), 1230 cm^−1^ (C–O ether), 1680, 1710, 1820 cm^−1^ (aromatic ring), 3300 cm^−1^ (O–H str) ^1^H NMR: (600 MHz, DMSO) δ 7.3 (2H, dd J = 8.3, 1.2 Hz), 7.3–7.5 (2H, m), 7.6 (1H, d, J = 1.7 Hz), 7.7 (1H, dd, J = 7.9, 1.1 Hz), 7.9 (1H, dd, J = 7.6, 1.6 Hz), 8.1 (2H, dd, J = 8.3, 2.1 Hz), 8.4 (1H, d, J = 1.7 Hz). ^13^C NMR (100 MHz, DMSO) δ (ppm) 100.9, 112.5, 126.3, 140.4, 156.0. MS m/z: calcd for C_18_H_11_BrN_4_O_3_ found 410.01 (M–H)^−^: 409.43.

**5k**: 2-(4-chlorophenyl)-6-(4-nitrophenoxy)-1*H*-imidazo[4,5-b]pyridine. Yield: 30%. M.P. 142 °C–144 °C. IR v = 1380 cm^−1^ (N–O str), 1180 cm^−1^ (C–O ether), 1690, 1850, 1730 cm^−1^ (aromatic ring), 3230 cm^−1^ (O–H str) ^1^H NMR: (600 MHz, DMSO) δ 7.3 (2H, dd, J = 8.3, 1.2 Hz), 7.6 (1H, d, J = 1.6 Hz), 7.7–7.8 (4H, m), 8.1 (2H, dd, J = 8.3, 2.1 Hz), 8.4 (1H, d, J = 1.6 Hz). ^13^C NMR (100 MHz, DMSO) δ (ppm) 100.9, 115.0, 128.0, 135.2, 151.2, 156.0. MS m/z: calcd for C_18_H_11_ClN_4_O_3_ found 366.05 (M–H)^−^: 365.04.

**5l**: 2-(4-methylphenyl)-6-(4-nitrophenoxy)-1*H*-imidazo[4,5-b]pyridine. Yield: 32%. M.P. 160 °C–162 °C. IR v = 1350 cm^−1^ (N–O str), 1240 cm^−1^ (C–O ether), 1650, 1710, 1810 cm^−1^ (aromatic ring), 3140 cm^−1^ (O–H str) ^1^H NMR: (600 MHz, DMSO) δ 2.3 (3H, s), 7.2–7.3 (4H, m), 7.66 (1H, d, J = 1.8 Hz), 7.9 (2H, dd, J = 7.9, 1.6 Hz), 8.1–8.1 (3H, m). ^13^C NMR (100 MHz, DMSO) δ (ppm) 100.9, 115.0, 129.3, 139.7, 140.4, 151.2, 156.0. MS m/z: calcd for C_19_H_14_N_4_O_3_ found 346.10 (M–H)^−^: 345.57.

**5m**: 5-methyl-2-[6-(4-nitrophenoxy)-1*H*-imidazo[4,5-b]pyridin-2-yl]phenol Yield: 28%. M.P. 142 °C–144 °C. IR v = 1410 cm^−1^ (N–O str), 1120 cm^−1^ (C–O ether), 1630, 1710, 1720 cm^−1^ (aromatic ring), 3410 cm^−1^ (O–H str) ^1^H NMR: (600 MHz, DMSO) δ 2.2 (3H, s), 7.2–7.2 (2H, m), 7.3 (2H, dd, J = 8.3, 1.2 Hz), 7.6 (1H, d, J = 1.7 Hz), 7.6 (1H, dd, J = 8.1 Hz), 8.1 (1H, d, J = 1.7 Hz), 8.1 (2H, dd, J = 8.3, 2.1 Hz). ^13^C NMR (100 MHz, DMSO) δ (ppm) 21.4, 100.9, 115.8, 127.8, 140.4, 152.3, 158.7. MS m/z: calcd for C_19_H_14_N_4_O_4_ found 362.10 (M–H)^−^: 361.15.

**5n**: 2-(2-methylphenyl)-6-(4-nitrophenoxy)-1*H*-imidazo[4,5-b]pyridine. Yield: 41%. M.P. 135 °C–137 °C. IR v = 1450 cm^−1^ (N–O str), 1140 cm^−1^ (C–O ether), 1730, 1810, 1730 cm^−1^ (aromatic ring), 3120 cm^−1^ (O–H str) ^1^H NMR: (600 MHz, DMSO) δ 2.2 (3H, s), 7.3 (2H, dd, J = 8.4, 1.2 Hz), 7.3 (1H, dd, J = 7.9, 1.1 Hz), 7.4–7.6 (2H, m), 7.6 (1H, d, J = 1.8 Hz), 7.7 (1H, dd, J = 7.7, 1.6 Hz), 8.1–8.1 (3H, m). ^13^C NMR (100 MHz, DMSO) δ (ppm) 19.8, 100.9, 124.4, 130.7, 140.4, 151.2, 156.0. MS m/z: calcd for C_19_H_14_N_4_O_3_ found 346.10 (M–H)^−^: 345.47.

**5o**: 2-(3-bromophenyl)-6-(4-nitrophenoxy)-1*H*-imidazo[4,5-b]pyridine. Yield: 30%. M.P. 166 °C–168 °C. IR v = 1490 cm^−1^ (N–O str), 1190 cm^−1^ (C–O ether), 1660, 1720, 1740 cm^−1^ (aromatic ring), 3340 cm^−1^ (O–H str) ^1^H NMR: (600 MHz, DMSO) δ 7.3 (2H, dd, J = 8.3, 1.2 Hz), 7.4 (1H, td, J = 8.0 Hz), 7.5 (1H, dd, J = 8.0, 1.6 Hz), 7.6 (1H, dd, J = 8.0, 1.5 Hz), 7.7 (1H, d, J = 1.6 Hz), 8.0 (1H, s, J = 1.5 Hz), 8.1 (2H, dd, J = 8.3, 2.1 Hz), 8.4 (1H, d, J = 1.6 Hz) ^13^C NMR (100 MHz, DMSO) δ (ppm) 100.9, 126.8, 135.2, 140.4, 151.5, 156.0. MS m/z: calcd for C_18_H_11_BrN_4_O_3_ found 410.0 (M–H)^−^: 409.45.

**5p**: 2-(3-methylphenyl)-6-(4-nitrophenoxy)-1*H*-imidazo[4,5-b]pyridine. Yield: 34%. M.P. 148 °C–150 °C. IR v = 1470 cm^−1^ (N–O str), 1250 cm^−1^ (C–O ether), 1670, 1750, 1860 cm^−1^ (aromatic ring), 3560 cm^−1^ (O–H str) ^1^H NMR: (600 MHz, DMSO) δ 2.2 (3H, s), 7.2–7.3 (3H, m), 7.5 (1H, dd, J = 7.9, 7.7 Hz), 7.6–7.7 (2H, m), 7.9 (1H, dd, J = 1.6, 1.5 Hz), 8.1–8.2 (3H, m). ^13^C NMR (100 MHz, DMSO) δ (ppm) 20.9, 100.9, 119.7, 135.2, 151.2, 151.5, 156.0. MS m/z: calcd for C_19_H_14_N_4_O_3_ found 346.10 (M–H)^−^: 345.50.

**5q**: 2-(3-methylphenyl)-6-(4-nitrophenoxy)-1*H*-imidazo[4,5-b]pyridine. Yield: 40%. M.P. 171 °C–173 °C. IR v = 1330 cm^−1^ (N–O str), 1160 cm^−1^ (C–O ether), 1680, 1650, 1820 cm^−1^ (aromatic ring), 3340 cm^−1^ (O–H str) ^1^H NMR: (600 MHz, DMSO) δ 2.2 (3H, s), 7.2–7.4 (3H, m), 7.4 (1H, dd, J = 7.9, 7.7 Hz), 7.6–7.6 (2H, m), 7.9 (1H, s, J = 1.5 Hz), 8.1–8.2 (3H, m). ^13^C NMR (100 MHz, DMSO) δ (ppm) 20.9, 100.9, 117.2, 128.4, 130.4, 140.4, 151.5, 156.0. MS m/z: calcd for C_19_H_14_N_4_O_3_ found 346.10 (M–H)^−^: 345.41.

**5r**: 5-methoxy-2-[6-(4-nitrophenoxy)-1*H*-imidazo[4,5-b]pyridin-2-yl]phenol Yield: 31%. M.P. 144 °C–146 °C. IR v = 1370 cm^−1^ (N–O str), 1260 cm^−1^ (C–O ether), 1720, 1710, 1690 cm^−1^ (aromatic ring), 3310 cm^−1^ (O–H str) ^1^H NMR: (600 MHz, DMSO) δ 3.8 (3H, s), 6.5 (1H, d, J = 1.6 Hz), 7.0 (1H, dd, J = 8.4, 1.6 Hz), 7.3 (2H, dd, J = 8.3, 1.3 Hz), 7.5–7.5 (2H, m), 8.1 (2H, dd, J = 8.3, 2.1 Hz), 8.3 (1H, d, J = 1.7 Hz). ^13^C NMR (100 MHz, DMSO) δ (ppm) 55.4, 100.6, 117.2, 135.2, 156.0, 161.8. MS m/z: calcd for C_19_H_14_N_4_O_5_ found 378.09 (M–H)^−^: 377.52.

**5s**: 2-(3,4-dimethoxyphenyl)-6-(4-nitrophenoxy)-1*H*-imidazo[4,5-b]pyridine. Yield: 38%. M.P. 166 °C–167 °C. IR v = 1350 cm^−1^ (N–O str), 1130 cm^−1^ (C–O ether), 1655, 1690, 1710 cm^−1^ (aromatic ring), 3320 cm^−1^ (O–H str) ^1^H NMR: (600 MHz, DMSO) δ 3.7 (3H, s), 3.8 (3H, s), 6.5 (1H, d, J = 6.2 Hz), 7.3 (2H, dd, J = 8.4, 1.4 Hz), 7.4 (1H, d, J = 1.7 Hz), 8.0 (1H, d, J = 1.7 Hz), 8.1 (2H, dd, J = 8.3, 2.1 Hz). ^13^C NMR (100 MHz, DMSO) δ (ppm) 56.1, 111.0, 119.7, 128.2, 140.4, 152.3, 156.0. MS m/z: calcd for C_20_H_16_N_4_O_5_ found 392.11 (M–H)^−^: 391.53.

**5t**: 2-(3-chlorophenyl)-6-(4-nitrophenoxy)-1*H*-imidazo[4,5-b]pyridine. Yield: 26%. M.P. 158 °C–160 °C. IR v = 1380 cm^−1^ (N–O str), 1220 cm^−1^ (C–O ether), 1665, 1780, 1670 cm^−1^ (aromatic ring), 3540 cm^−1^ (O–H str) ^1^H NMR: (600 MHz, DMSO) δ 7.3 (2H, dd, J = 8.3, 1.2 Hz), 7.4–7.5 (2H, m), 7.6 (1H, dd, J = 8.0, 1.6 Hz), 7.7 (1H, d, J = 1.6 Hz), 7.8 (1H, s, J = 1.5 Hz), 8.1 (2H, dd, J = 8.3, 2.1 Hz), 8.4 (1H, d, J = 1.6 Hz). ^13^C NMR (100 MHz, DMSO) δ (ppm) 100.9, 119.7, 126.8, 129.5, 151.7, 156.0. MS m/z: calcd for C_18_H_11_ClN_4_O_3_ found 366.05 (M–H)^−^: 365.55.

**5u**: 2-(3-bromophenyl)-6-(4-nitrophenoxy)-1*H*-imidazo[4,5-b]pyridine. Yield: 41%. M.P. 160 °C–162 °C. IR v = 1330 cm^−1^ (N–O str), 1280 cm^−1^ (C–O ether), 1620, 1830, 1790 cm^−1^ (aromatic ring), 3130 cm^−1^ (O–H str) ^1^H NMR: (600 MHz, DMSO) δ 7.3 (2H, dd, J = 8.3, 1.2 Hz), 7.6 (1H, d, J = 1.7 Hz), 7.7 (2H, dd, J = 8.2, 1.6 Hz), 7.8 (2H, dd, J = 8.2, 1.6 Hz), 8.1 (2H, dd, J = 8.3, 2.1 Hz), 8.4 (1H, d, J = 1.7 Hz). ^13^C NMR (100 MHz, DMSO) δ (ppm) 100.9, 119.7, 128.3, 135.2, 151.2, 156.0. MS m/z: calcd for C_18_H_11_BrN_4_O_3_ found 410.0 (M–H)^−^: 409.46.

**5v**: 6-(4-nitrophenoxy)-2-(3-nitrophenyl)-1*H*-imidazo[4,5-b]pyridine. Yield: 32%. M.P. 128 °C–130 °C. IR v = 1340 cm^−1^ (N–O str), 1240 cm^−1^ (C–O ether), 1680, 1840, 1770 cm^−1^ (aromatic ring), 3210 cm^−1^ (O–H str) ^1^H NMR: (600 MHz, DMSO) δ 7.3 (2H, dd, J = 8.4, 1.4 Hz), 7.6 (1H, dd, J = 8.6, 8.0 Hz), 7.7 (1H, d, J = 1.6 Hz), 8.1 (2H, dd, J = 8.4, 2.1 Hz), 8.3 (1H, dd, J = 8.0, 1.9 Hz), 8.5 (1H, dd, J = 8.6, 1.9 Hz), 8.6 (1H, d, J = 1.6 Hz), 8.9 (1H, dd, J = 1.6, 1.5 Hz). ^13^C NMR (100 MHz, DMSO) δ (ppm) 100.9, 117.8, 135.2, 151.2, 156.0. MS m/z: calcd for C_18_H_11_N_5_O_5_ found 377.07 (M–H)^−^: 376.45.

**5w**: 2-(2-fluorophenyl)-6-(4-nitrophenoxy)-1*H*-imidazo[4,5-b]pyridine. Yield: 29%. M.P. 140 °C–142 °C. IR v = 1390 cm^−1^ (c), 1240 cm^−1^ (C–O ether), 1630, 1840, 1690 cm^−1^ (aromatic ring), 3310 cm^−1^ (O–H str) ^1^H NMR: (600 MHz, DMSO) δ 7.3 (2H, dd, J = 8.3, 1.4 Hz), 7.3–7.5 (3H, m), 7.6 (1H, d, J = 1.7 Hz), 7.9 (1H, dd, J = 7.6, 1.6 Hz), 8.1 (2H, dd, J = 8.3, 2.1 Hz), 8.4 (1H, d, J = 1.7 Hz). ^13^C NMR (100 MHz, DMSO) δ (ppm) 100.9, 115.0, 130.6, 151.2, 160.4. MS m/z: calcd for C_18_H_11_FN_4_O_3_ found 350.08 (M–H)^−^: 349.53.

### Biological evaluation

All synthesised compounds were subjected to anti-tubercular activity against the pathogenic strain for *Mycobacterium tuberculosis* (H_37_Rv) ATCC 27294. *M. tuberculosis* (Mtb) H_37_Rv ATCC 27294 used for determination of MIC was cultured according to method reported previously by Martin et al. [42]. A single seed lot maintained at − 70 °C was used for obtaining the inoculums for all the experiments. The bacteria was grown in roller bottles containing Middlebrook 7H9 broth supplemented with 0.2% glycerol, 0.05% Tween 80 (Sigma), and 10% albumin dextrose catalase obtained from Difco Laboratories, USA, at 37 °C for 7–10 days. The cell colony was harvested by carrying out centrifugation then it was washed twice in 7H9 broth again it was suspended in fresh 7H9 broth. Several aliquots of 0.5 ml were dispensed and the seed lots of suspension was stored at − 70 °C for further use. To test the viability of prepared culture one of the vial was thawed and plate cultured to determine the colony forming unit (CFU). For compounds **5a**–**w**, stock solutions and dilutions were prepared, all test compound stocks and dilutions were prepared in DMSO. Minimum Inhibitory Concentrations (MIC) of all test compounds were determined in Middlebrook 7H9 broth by the standard microdilution method. In a 384 well plate 1 ml of serial two-fold dilutions of test compound was poured in concentration range of 100 µM–0.19 µM. The control wells contained media and culture controls only; Isoniazid was used as standard reference for the assay. As per the reported method, 40 ml (3–7 × 105 CFU/ml) of the bacterial culture was added to all the wells. Only the control wells were devoid of culture. The plates were incubated at 37 °C for 5 days packed in gas permeable polythene bags. After the completion of incubation period, each well was introduced with a freshly prepared 1:1 mixture of Resazurin (0.02% in water), and 10% Tween 80 with 8 ml in quantity. It was understood that change in colour indicates growth or inhibition, if the colour of solution in well changes to blue then it is assumed as inhibition and if changes to pink then growth of the culture. To determine this change all the plates were again incubated for 24 h at 37 °C and then the change in each well was observed. A concentration at which change of colour from blue to pink in inhibited shall be considered as the MIC. Solutions from all the wells were studied for their absorbance at 575 nm and 610 nm then ratio was calculated, an 80% inhibition was considered as MIC. The minimum bactericidal concentration (MBC) is the lowest concentration of an antibacterial agent required to kill the bacteria under study. Aliquots from sample wells (MIC and higher) from the MIC plates were diluted 1:10 and sub cultured on 7H10 agar plates. These were incubated at 37 °C for 3–4 weeks (without test compounds), CFU was studied. The lowest concentration of test compound that resulted in a reduction of about two log_10_ CFU from the initial unit was considered as MBC.

### Molecular docking

Crystal structure of protein (PDB code: 4KW5) was obtained from RCSB protein Data Bank. The receptor molecule was refined using protein preparation wizard module on the maestro molecular modeling interphase, Schrodinger software. Ligands-glycerol, imidazole, FAD and ethyl ({2-[(1,3-benzothiazol-2-ylcarbonyl)amino]thiophen-3-yl}carbonyl)carbamate were already present within the receptor in bound form. All ligands were removed except ethyl ({2-[(1,3-benzothiazol-2-ylcarbonyl)amino]thiophen-3-yl}carbonyl)carbamate to allow for docking protocol [43–50]. For this study, all the ligands were prepared and docked for in flexible docking mode and atoms located within a range of 3.0 Å from the amino acid residues were selected in the active site. The docking calculations and energy minimization were set in the ligand docking module, most of the parameters were set default. This cavity consisted of amino acid residues Lys134, Tyr314, Ser228, Lys367, Asn385, Gln336, His132, Val365, Gln334, Cys387, Tyr60, Lys418. This cavity was selected on the basis of reported crystal structure of lead molecule ethyl ({2-[(1,3-benzothiazol-2-yl carboxyl)amino]thiophen-3-yl}carbonyl) carbamate.

## Results and discussion

### Chemistry

The process of four step sequence was initiated with acetylation of 5,6-diaminopyridine-3-ol **1** on reaction using acetic anhydride to form compound **2**. Detail reaction data is not mentioned for this step in the manuscript as this is well known step in organic synthesis. Further, compound **2** was treated with potassium carbonate diluted in dimethyl formamide and latter with *p*-chloronitrobenzene to form ether linkage **3**. The reaction sequence was continued with process of deacetylation by refluxing with 70% sulphuric acid and 10% sodium hydroxide for 20–30 min to obtained compound **4**. Compound **4** was treated with various substituted aryl aldehydes to get desired derivatives. Reaction steps were monitored by TLC. Spectroscopic studies were carried out for all the synthesized compounds including intermediates. The IR spectrum showed absorption bands at 1540 cm^−1^ (N–O str) confirms the presence of nitro group, 1180 cm^−1^ (C–O str) confirms the ether linkage, bands at 1480 cm^−1^, 1550 cm^−1^, 1690 cm^−1^, 1740 cm^−1^ indicates the presence of aromatic rings. ^1^H NMR study displays the protons between δ 7.3 and 8.3 belongs to aromatic ring of imidazopyridine. The ^13^C NMR studies indicate the aromatic carbons. The compounds were also confirmed by mass analysis.

### Molecular docking

**Fig. 1:**
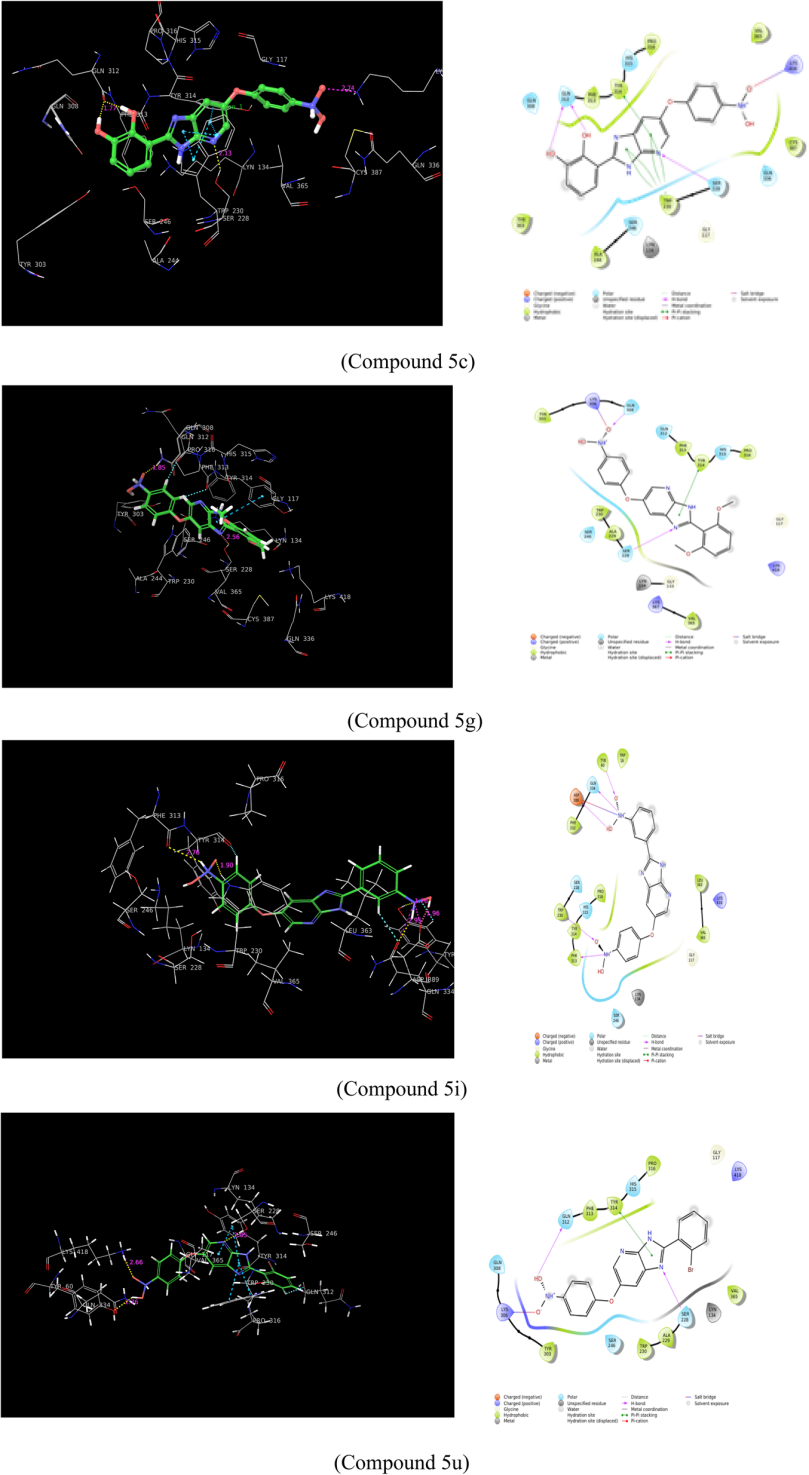
Binding model of compounds **5c**, **5g**, **5i** and **5u** with DprE1 target cavity. It represents hydrogen bonds, hydrophobic interactions and pi-pi interactions

The molecular docking study was carried out to uncover the best possible binding modes for newly synthesized derivatives with the enzyme (DprE1). The docking simulations were carried out by Glide docking tool of Maestro molecular modeling interphase (Schrodinger, USA). The receptor employed here was specifically DprE1 (PDB code: 4KW5) obtained from RCSB Protein Data Bank (RCSB-PDB). The initial crystal structure consisted of the bound ligand, it was removed and the missing loops were added. The docking scores of all the compounds were presented in (Table 2). The interacting amino acid residues were identified as Tyr 314, Lyn134, Trp230, Gln 334, Asp389, Phe313, Ser228, Gln312, Lys418, Trp320, Tyr60. The binding modes of the four compounds are presented in (Fig. 1). Imidazopyridine nucleus of compound **5c** has shown number of overlaps in pi–pi stacking with Trp230, and Tyr314 also H-bond was observed between nitrogen of pyridine of Imidazopyridine nucleus and Ser228. Both the hydroxyl groups on substituted phenyl ring shows interaction with Gln312. Nitro on phenyl ring connected to Imidazopyridine nucleus by ether linkage shows interaction with Lys418. In compound **5g**, nitrogen of Imidazopyridine ring forms hydrogen bond with Ser 228. Tyr314 also shows pi–pi stacking with Imidazopyridine nucleus. Compound **5i** emphasizes on interactions of oxygen, proton of nitro group on phenyl ring connected by ether linkage with Trp230, Phe313 respectively where as two oxygen and a proton from nitro group on substituted phenyl ring forms H-bonds with Tyr60, Asp389 and Gln334 respectivey, proton also forms overlapping salt bridge with Asp389. In compound **5u**, nitrogen from Imidazopyridine ring forms H-bond with Ser228 and pi–pi stacking with Tyr314, oxygen of phenyl substituted nitro group has shown interaction with Gln 312. Interactions produced by these molecules are quite similar to the lead molecule TCA1, this directs that a substitution with Imidazopyridine nucleus may contribute towards the DprE1 selectivity leading to development of the target specific lead molecules for this series forming potent antitubercular agents.

**Table 2:** Data of the in vitro studies for *M. tuberculosis* (H_37_Rv) and docking score of synthesized compounds

Compound ID	Antitubercular activity MIC (μmol/L) on H_37_RV	Docking score	Compound ID	Antitubercular activity MIC (μmol/L) on H_37_RV	Docking score
**5a**	1.2	− 7.234	**5m**	1.7	− 6.964
**5b**	1.5	− 7.140	**5n**	1.2	− 5.761
**5c**	0.6	− 7.500	**5o**	1.1	− 6.657
**5d**	1.1	− 7.400	**5p**	1.5	− 6.193
**5e**	1.7	− 6.695	**5q**	1.4	− 6.186
**5f**	2.3	− 7.081	**5r**	1.6	− 7.084
**5g**	0.5	− 7.698	**5s**	1.4	− 5.793
**5h**	1.1	− 7.286	**5t**	1.8	− 5.761
**5i**	0.8	− 8.825	**5u**	0.7	− 8.213
**5j**	2.1	− 7.611	**5v**	2.6	− 6.657
**5k**	1.9	− 6.685	**5w**	1.0	− 5.836
**5l**	1.3	− 5.761	Isoniazid	0.3	− 7.328

### Antitubercular activity

In vitro anti-tubercular studies for determination of minimum inhibitory concentration (MIC) and minimal bactericidal concentration (MBC) The in vitro studies were carried out on *M. tuberculosis* H_37_Rv (ATCC 27294) strain to determine MIC of test compounds with Isoniazid as standard reference. Microbial culture was developed on Middlebrook 7H9 broth supplemented with 0.2% glycerol, 0.05% Tween 80 (Sigma), and 10% albumin dextrose catalase. The test compounds were prepared as stock and dilutions in DMSO and MIC was determined by microdilution technique. After the incubation period of culture in presence or absence of test compounds, the viability of bacteria was determined by observing the colour change from blue to pink of resazurin mixture which acts as indicator of the inhibitory activity and potency. It was found that compounds **5c**, **5g**, **5i** and **5u** exhibited MIC between 0.5 and 0.8 µM which is found very close to the standard reference Isoniazid with MIC of 0.3 µM. The compounds with good MIC were found to be substituted with nitro, methoxy, hydroxyl and halogens like fluorine, chlorine, bromine. Earlier it was reported that nitro group containing compounds inhibit DprE1 selectively due to conversion of the nitro to reduce form and then its interaction with Cys387 residue. Here, we didn’t observed any interaction of synthesized compounds with Cys387 but most of compounds exhibited good docking score with better In vitro antitubercular activity. Furthermore, we have plan to test the compounds with subject to enzyme specific DprE1 inhibitory activity.

## Conclusion

We have reported a series of 6-(4-nitrophenoxy)-1*H*-imidazo[4,5-b]pyridine Derivatives **5a**–**w**. Newly synthesized compounds were tested for their In vitro antitubercular activity on the virulent strain H_37_RV of *M. tuberculosis*. Few compounds have shown attractive antitubercular activity, among the active compounds, **5c**, **5g**, **5i** and **5v** have shown good potency towards *M. tuberculosis* strain. Molecular docking studies were also carried out using the reported crystal structure of DprE1, we studied flexible binding modes for the synthesized compounds in comparison with the cocrystal reference molecules TCA1 and BTZ043. Interestingly, same compounds (**5c**, **5g**, **5i** and **5v**) were come up with excellent docking score. Knowledge from the molecular docking studies emphasize that further modifications are also possible in the series of molecules to develop better compounds for potential DprE1 inhibitory activity. Previously, it was reported that nitro group gets reduced and forms adduct with Cys387 to exhibit DprE1 inhibitory activity. Current molecular docking studies strikes on interactions of synthesized chemical structures with various amino acid residues but does not showed any interaction with Cys387 residue but shown excellent docking score. These compounds may exhibit DprE1 inhibitory activity. This information on ligand binding in active site from crystal structure can be utilised for further medicinal chemistry efforts to study enzyme specific inhibition study (Additional file 1).

## Additional file

**Additional file 1.** ^1^H and ^13^C NMR spectra of all newly synthesized (5a–w) compounds.
