# Supplementary material for: Synthesis, biological evaluation and molecular docking studies of 6-(4-nitrophenoxy)-1H-imidazo[4,5-b]pyridine derivatives as novel antitubercular agents: future DprE1 inhibitors
Source: Chem Cent J. 2018 Dec 19;12:138. doi: 10.1186/s13065-018-0515-1 (PMC6768143; doi:10.1186/s13065-018-0515-1)
Supplement: Supplementary file 1 — Additional file 1. 1H and 13C NMR spectra of all newly synthesized (5a–w) compounds. [file 13065_2018_515_MOESM1_ESM.doc]

1H NMR Data of Newly Synthesized Compounds

5a

5b

5c

5d

5e

5f

5g

5h

5i

5j

5k

5l

5m

5n

5o

5p

5q

5r

5s

5t

5u

5v

5w

13C NMR Data of Newly Synthesized Compounds

5a

5b

5c

5d

5e

5f

5g

5h

5i

5j

5k

5l

5m

5n

5o

5p

5q

5r

5s

5t

5u

5v

5w
